# Churches and Charity Funds

**Published:** 1917-01-27

**Authors:** J. H. Wesley Kane

**Affiliations:** Vicar of Perry Hill and Catford Bridge, S.E. The Vicarage, Perry Hill, London, S.E.


### THE EDITOR'S LETTER-BOX.

### Churches and Charity Funds.

*To the Editor of* THE HOSPITAL.

SIR,—On the whole I should say that few churches deduct an average from their collections on Hospital Sunday. I am not an offender, so I write with a conscience void of offence; indeed, for twenty years I have fought on your side, and as a rule I send out at least 1,500 letters every June in the cause. But as to surplus collections in other causes it is a case of "better half a loaf than no bread." Hospitals and missions are two great crying needs and should be wholeheartedly supported, but there are one hundred and one other causes so deserving and so urgent that come under the heading "Better half a loaf," etc. Most of the suburban churches are without endowment of any kind, the multifarious charities and organisations have to be maintained from the collections. Charity begins at home, but it must not end there. An outside cause is in urgent need, the clergy and churchwardens are anxious to help, but it is a case of a surplus collection or nothing. As a rule people set aside a fixed sum Sunday by Sunday for the collection; this is probably doubled for the surplus collection, with the result that if the usual collection for the Sunday is £8 the wardens place it at the lowest figure, say £6 10s., and all over and above this sum is given to the good cause. Some churches hold alms-bags at the door so that at the close of the service the congregation have the opportunity of giving what they desire. They have already given to the offertory, which is voluntary and can be withheld for the cause that has been pleaded for. I have, however, generally found that this course is not nearly so successful as the surplus collection. Your article will probably do more harm than good. I wheedle surplus collection after surplus collection out of my wardens, not a penny of which I would obtain from outside charities if the whole collections were given. The churchwardens would refuse to face the Easter Vestry or continue their work with financial disaster facing them. Just now everybody is giving to the Red Cross through the Red Cross organisation in their respective parishes—*i.e.*, everybody who can—month in, month out, and it is difficult for those churches whose entire upkeep depends upon the weekly offertory, already much depleted by the war, to give away whole collections when they are already deeply in debt. I speak from full knowledge, and of that which I know; and though an ardent advocate for whole collections, I say from my heart, "Half a loaf is better than no bread."—I am, Sir, yours very truly, J. H. WESLEY KANE,
Vicar of Perry Hill and Catford Bridge, S.E.

The Vicarage, Perry Hill, London, S.E.,
January 17, 1917.

